# Civilization and Health

**Published:** 1880-02

**Authors:** 


### CIVILIZATION AND HEALTH.

THE past history of nations is conclusive as to one point, that prosperity begets refinement, luxury, disease and ruin. Is this a necessary result? Will this great and prosperous country, with its daily developing improvements, tending to the reduction and perfection of labor, as well as to the conveniences and comforts of life, eventually fall into effeminacy and extinction? We utter a decisive negative. There are two kinds of civilization, the ignorant and the educated. Of two families in all respects equal, having at their command every modern convenience, one will live in high health, and in the steady enjoyment of the blessings of life until an honorable old age, while the other will as certainly fade away, the children perishing first, and last of all the parents; and even they, long before the attainment of three score years and ten; their very names blotted from social memory! This wide difference is the direct result of the manner of life of the respective families, one having lived rationally, having lived up to the laws of our being, the other having wholly neglected them; the latter dying off prematurely, have cut off the race of effeminate imbeciles while the former have handed down to society the bequest of healthful constitutions. Thus we perceive, that educated civilization will perpetuate a nationality, while an uneducated one destroys it.

But in the fierce race which the masses run for pleasure, wealth, fame, is there any probability of inducing any great number to stop awhile in their course, and learn something of a true life? There are a few such in all communities; and as these leave seed, while the others leave none, the inequality will rapidly diminish; thus it is, that within a hundred years, the average of human life has increased all over the world, but more largely in its most civilized portions. The investigations and teachings of the true laws of our being have been confined to the medical profession, and they have been pursued with a diligence, and a self-denial, practised by no class of men on the habitable globe; because, for the most part, these investigations have been made under circumstances of animal and human suffering, of squalor, disgust and horror often, which, to any other than a trained medical mind, would have been impossible of endurance.

We may say with great truth that the materiel glory, permanence and power of any community consists in the physical vigor of the individual men and women who compose it; for physical perfection gives mental energy, and mental health. An exemplification of this important truth is found in the stability of every thing English, and the evanescent state of every thing French. We believe that physical perfection begets mental vigor, and that in turn, by appropriate tuitions, begets moral power, and that this combination makes the perfect man.

Many persons are frightened away by the mere mention of "*Living up to the laws of our being*," and at once begin to think of something painfully abstruse, or laboriously indefinite; an image of feeling after something in a fog at once arises before their mind, and anon come spectres of self-denial, starvation, physic and pills *ad infinitum.*

In all investigations, it is best to clear away the rubbish first and look for some foundation stones; to ferret out some first principles, some elementary ideas, which must in the very nature of things be few and well defined, and consequently, as facile of remembrance, as they are practicable in their application.

The Holy Scriptures, with beautiful exactness, declared four thousand years ago, what the scientific investigations of subsequent ages have steadily confirmed, that *the blood is the life* of all animal being, it is the blood which originates, governs and completes every vital power in the whole machinery of man; consequently, perfect health is only to be secured by maintain ing the blood in its natural state. The researches of the lights of our profession have established the facts, that this natural state of the blood comprehends a four-fold development.

1st. The Organic element, or *Chylid.*

2d. The Coloring element, or *Hæmatid.*

3d. The Animal element, or *Lymphid.*

4th. The Fluid element, or *Liquor Sanguinis.*

In a few hours after food is eaten, it is converted into a whitish, sweetish, thickish fluid, whatever may be the nature of the food; but in it are found innumerable little globules which are called "*Chylids;*" these globules consist of a little bladder or cell, in which is an atom, called an egg, the cell being a boat floating about in the chyle, the atom is its freight, which as it passes along, becomes a living thing, as an egg becomes a chick; but being quickened into life it changes into a reddish color, and takes another name in its new and living nature, and is called a "*Hæmatid.*" This wonderful change from dead food to living existence, owes its origin to that equal Power which made all worlds. These animalcule *Hæmatids* are so diminutive that a small box, an inch deep, an inch broad and an inch long, will hold more than a hundred thousand millions of them. These *Hæmatids* are the foundation of all health and life; if they are transported in their little boats in unimpaired vigor to the different parts of the body, those parts grow with the same life and health which these Hæmatids have, but if injured in their transmission in any way, the part of the body to which they go is inevitably injured—becomes diseased. Our next step then is to inquire, taking it for granted that digestion is good, what circumstances in practical life have the effect to injure these new-born voyagers?

The blood of a vigorous man, on the instant of being drawn, is just as full of life as our own great Broadway on any sunny afternoon; it is this life which gives the blood its solidity, or more properly, its thickness. When a person dies from using chloroform, the blood is as liquid almost as water; it does not coagulate, become thick and clotted as the blood does from natural or other forms of death; on examining into the cause, it is discovered that of all the millions of *Hæmatids*, not one single one is alive, for the little cell boat has been dissolved, and its occupant has perished; the poison from the bite of venomous snakes has the same effect.

it is found also, that when a person dies by breathing the fumes of charcoal, or breathing carbonic acid gas in any other form, every single Hæmetid is found dead, asphyxiated, just as the subject was. If then, breathing carbonic acid gas kills the *Hœmatids*, they carrying none of their life to the different parts of the body, the man himself just as certainly dies, because his supply of life is cut off, and if for any single minute this living freight of Hæmetids is arrested, that minute we die.

A little reflection here will suggest one of the most important principles connected with human health—that is to say, out-door air has no carbonic acid gas, hence they who breathe it always revel in glorious health.

If again, pure carbonic acid gas as certainly kills a man in a short time as the breathing of chloroform or the poison of an adder, by killing the Hæmetids, so any air breathed, in proportion as it is impregnated with carbonic acid gas, will do violence to the life of the Hæmatids. But a man in sleeping, not only breathes out carbonic acid gas, but converts the air in a close room into carbonic acid gas, and the smaller the room the sooner will that conversion be made, and the closer the room the more perfect will be that conversion.

It will be thus seen that it is an utter impossibility for any one to sleep for a single night in a room with windows and doors closed without inflicting death at its birth to that which otherwise would have given to the body vigor, health and life. And although the mischief is not made apparent by the death of the individual next morning, that mischief is not the less real, although it is less extensive, and its ill results are sooner or later inevitable. Within a year a ship was undergoing an examination in a dry dock, and at a certain point its bottom for a few inches square, was found to be not thicker than a piece of paper. On examination, it was ascertained that a small pebble was lodged in the space between the plank which faced the water and that which made the inner floor of the vessel; it had been there for two years, and with every motion of that vessel on its billowy home that little pebble also moved, and in its motion wore away some of the timber; too small it may be for detection by any ordinary microscope, but in the course of a year it was enough to wear away an inch of solid timber, and in the second year, nearly two inches more, for, with the increase of room which it made for itself, there was an increase of momentum, and consequent wear. Because the captain of that vessel was ignorant of that imprisoned pebble, and because he saw no indication of its destructive influences, those influences were not the less real, and not the less certain of terrible disaster, but for the fortunate discovery. Thus it is with human life and health, the breathing of a vitiated atmosphere, whether in close and small rooms or large and close bedrooms, or in family rooms over cellars without ceilings, whose noisome odors rise incessantly day and night to the upper portions of the buildings—the fumes from decayed vegetables, barrels and boxes sodden with dampness, which have not seen the light of the sun for years, saying nothing of old bones, rags, brooms and various other things for which the cellar is used as a common receptacle; or whether these miasms and malarias are generated in dirty back yards, or piles of sweepings heaped up under stairs or in closets or dark corners, or from livery stables, or cow houses, or pig-pens, or butcher stalls, or vegetable markets—we repeat, the breathing of such or other vitiated atmospheres does, by an immutable law of nature, bring injury to the system with the same certainty that gravity will affect a projected feather, or cannon ball or mountain.

These are truths which every person should know for himself and should teach to his children from their earliest years, for it is only by the diffusion and practice of knowledge like this, that we can ever hope to see a healthy offspring and to enjoy, not only with impunity, but with advantage, all that is meant by the term "*modern conveniences.*"

We fear that this article is already too long to be read by the many, or to be copied in our weekly newspapers, which so regularly visit almost every family in this broad land, but we trust it will be copied, for it is by the incessant and wide and repeated instillation of sentiments like these, that we expect to build up a public sentiment which will appreciate the high and enduring advantages resulting from a habitual breathing of a pure atmosphere.

